# Presence of porcine cytomegalovirus, a porcine roseolovirus, in wild boars in Italy and Germany

**DOI:** 10.1007/s00705-022-05690-6

**Published:** 2023-01-07

**Authors:** Sabrina Hansen, Maria Luisa Menandro, Giovanni Franzo, Ludwig Krabben, Stephen F. Marino, Benedikt Kaufer, Joachim Denner

**Affiliations:** 1 Institute of Virology, Free University Berlin, 14163 Berlin, Germany; 2 Department of Animal Medicine, Production and Health (MAPS), University of Padua, 35020 Legnaro, Italy; 3 Parasites in Foodstuffs, Department of Biological Safety, Unit Diagnostics, German Federal Institute for Risk Assessment, 10589 Berlin, Germany

## Abstract

Porcine cytomegalovirus (PCMV), a porcine roseolovirus (PRV) that is closely related to human herpesviruses 6 and 7, is commonly found in commercial pigs. PCMV/PRV is important in xenotransplantation, because in preclinical trials in which pig organs were transplanted into non-human primates, transmission of PCMV/PRV was shown to be associated with significantly reduced survival of the xenotransplants. PCMV/PRV was also transmitted in the first transplantation of a pig heart into a human patient worldwide and apparently contributed to the death of the patient. The prevalence of PCMV/PRV in wild boars is largely unknown. In this study, we screened wild boars from several areas of northern Italy and Germany to test for the presence of PCMV/PRV using PCR-based and Western blot assays. By Western blot analysis, 54% and 82% of Italian and German wild boars, respectively, were found to be PCMV/PRV positive, while 36% and 60%, respectively, tested positive by real-time polymerase chain reaction (PCR). These data indicate that the virus is common in German and Italian wild boars and that the Western blot assay detected a PCMV/PRV infection more often than did real-time PCR. The data also indicate that pigs raised for xenotransplantation should be protected from contact with materials from wild boars and commercial pigs.

**Supplementary Information:**

The online version contains supplementary material available at 10.1007/s00705-022-05690-6.

## Introduction

Porcine cytomegalovirus (PCMV) has a misleading name. It is a herpesvirus that is closely related to human herpesviruses 6A, 6B, and 7 (HHV-6A, -6B, and -7), but it is only distantly related to human cytomegalovirus (HCMV, also called human herpesvirus 5, HHV-5) [1, 2]. It is, rather, a roseolovirus and should, more precisely, be called PCMV/porcine roseolovirus (PCMV/PRV) to indicate the difference. According to the International Committee on Taxonomy of Viruses (ICTV), it is also called suid betaherpesvirus 2 or suid herpesvirus 2 (SuHV2) [3]. The appearance of cytomegalic cells with characteristic basophilic intranuclear inclusion bodies in the mucosal glands of turbinates of pigs was likely the reason for the misleading name [4]. Pulmonary macrophage cultures derived from three- to five-week-old piglets have been shown to be the most sensitive system for both primary isolation and propagation of this virus [5]. Cytomegaly and intranuclear inclusions developed 11 to 14 days after inoculation [6]. Like HHV-6A, -6B, and 7 and another related roseolovirus, murine roseolovirus (MRV), PCMV/PRV is present throughout the world, and nearly 90% of the animals in some pig herds are infected [7–9]. Western blot assays detected PCMV/PRV-specific antibodies in most of the animals from a German slaughterhouse, indicating viral infection [10]. Using a newly developed one-tube nested real-time PCR assay, 38.6% of Chinese pigs were found to be PCMV positive [11]. Infection usually occurs early in life [12, 13].

In the context of xenotransplantation, PCMV/PRV is the second known zoonotic (disease-inducing) pig virus after hepatitis virus E genotype 3 (HEVgt3 or HEV-3) [14]. PCMV/PRV has been shown to be responsible for a drastically reduced survival time of pig kidney or heart xenotransplants in non-human primates [15–21]. For example, the survival time of pig hearts orthotopically transplanted into baboons was less than 30 days when they were infected with PCMV/PRV, whereas the survival time of virus-free pig hearts was up to 195 days [19]. PCMV/PRV was also transmitted during the first transplantation of a pig heart to a human in Baltimore and apparently contributed to the death of the patient [22]. The method used for testing the donor animal was not suitable.

Wild boars can serve as reservoirs for a number of bacteria, viruses, and parasites that are transmissible to humans and domestic animals through direct interaction with the animals, through contaminated food, or indirectly through contamination in the environment [23, 24]. Numerous viruses have been detected in European wild boars, such as porcine circovirus 2 (PCV2) in Italy [25, 26], Ukraine [27], and Portugal [28], porcine circovirus 3 (PCV3) in Germany [29], Spain [30], Italy [25, 26, 31], and Austria [32], HEV-3 in Spain [33, 34], Germany [35, 36], Italy [37–39], Poland [40, 41], Bulgaria [42], and Serbia [43], porcine lymphotropic herpes viruses 1, 2, and 3 (PLHV-1, -2, -3) in Austria [32], porcine parvovirus 1 (PPV1) in Italy [44], and suid herpes virus 1 (SuHV-1 or pseudorabies virus, PrV) in Italy [45], Slovenia [46], Switzerland [47], and Germany [48, 49].

In contrast, the prevalence of PCMV/PRV in wild boars is not well studied. The first time PCMV/PRV was detected in a wild boar was in Japan in 2013 [50]. PCMV/PRV has also been found in wild boars in Argentina and Russia [51, 52]; however, there are no reports on PCMV/PRV in wild boars in Western Europe.

In order to fill the gap in our knowledge concerning PCMV/PRV in wild boars in Europe, we used real-time PCR and Western blot analysis to determine, for the first time, the prevalence of PCMV/PRV in two European countries: Italy and Germany.

## Materials and methods

### Animals

Sera from 74 wild boars from different locations in northern Italy – 50 from the Euganean Hills, 10 from the Veneto Alps, 12 from the Friuli Venezia Giulia Alps, and two from the Lombardian Alps (Table 1, Supplementary Fig. S1) were collected in the years 2017, 2018, and 2020, most of them in 2017 and 2018. The age of the animals was estimated by dentition characteristics and ranged from two months to 36 months. After that age, dentition is no longer an accurate indicator of an animal's age, and therefore, older pigs were classified in a single category. Most of the animals were approximately 12 months old. The animals were then divided into two categories for the analysis: Up to 22 months (54 pigs) and older that 22 months (18 pigs). For two animals, the age was not established. The weight of the animals ranged from 11 to 96 kg.

**Table 1:** Results of the testing of wild boars from different locations in Italy

Location	All animals		Up to 22 months		Older than 22 months	
	Number Western blot positive /number tested (%)	Number real-time PCR positive/number tested (%)	Number Western blot positive/number tested (%)	Number real-time PCR positive/number tested (%)	Number Western blot positive/number tested (%)	Number real-time PCR positive/number tested (%)
Euganean Hills	26/50 (52)	15/50 (30)	19/38 (50)	11/38 (29)	4/12 (33)	4/12 (33)
Veneto Alps	7/10 (70)	8/10 (80)	3/6 (50)	4/6 (67)	4/4 (100)	4/4 (100)
Friuli Venezia Giulia Alps	3/12 (25)	3/12 (25)	3/10 (30)	3/10 (30)	0/2 (0)	0/2 (0)
Lombardian Alps	1/2 (50)	1/2 (50)	-	-	-	-
Total	40/74 (54)	27/74 (36)	25/54 (46)	16/54 (30)	8/18 (44)	8/18 (44)

Sera from 50 German wild boars were collected at different locations in the state of Brandenburg in northeastern Germany (Table 2, Supplementary Fig. S2). Most of these sera were collected in autumn 2021, but some were collected in 2022. Forty-six of the 50 animals were female. The age of the animals was estimated based on their size and weight: 13 animals were 24 months old, 16 were 12 months old, and 21were less than twelve months old.

**Table 2:** Results of the testing of wild boars from different locations in Germany

Location	Number of Western blot positive/number tested (%)	Number of real-time PCR positive/number tested (%)
Wittstock	11/12 (92)	11/12 (92)
Döberitzer Heide	7/7 (100)	4/7 (57)
Lehnin	4/5 (80)	4/5 (80)
Rauen-Zerwelin	8/8 (100)	6/8 (75)
Ihlandsee-Wilkendorf	0/1 (0)	0/1 (0)
Lehnitz	0/1 (0)	0/1 (0)
Horstwalde	3/3 (100)	2/3 (67)
Niederlehme-West	3/6 (50)	1/6 (17)
Grubenmühle/Storkow	3/5 (60)	1/5 (20)
Rüthnicker Heide	1/1 (100)	1/1 (100)
Güterfelde	1/1 (100)	0/1 (0)
Total	41/50 (82)	30/50 (60)

### DNA extraction

DNA/RNA was purified from the samples using an innuPREP Virus DNA/RNA Kit (Analytik Jena, Jena, Germany) according to the manufacturer´s instructions. RNA/DNA was eluted in 60 µl of nuclease-free water. The samples were stored at -20°C until further processing.

### Real-time polymerase chain reaction (PCR)

The detection of PCMV/PRV was performed using a real-time PCR assay with specific primers and a probe [53, 54] (Table 3) as described previously [15, 55, 56]. All experiments were performed using a SensiFAST Probe No-ROX Kit (Meridian Bioscience, Cincinnati, OH, USA) and a qTOWER3 G qPCR cycler (Analytik Jena, Jena, Germany). All assays were performed in a duplex real-time PCR format with a specific primer-probe mixture (Table 3) and using the porcine glyceraldehyde-3-phosphate dehydrogenase (pGAPDH) gene as a reference. A reaction volume of 20 µl was prepared containing 1.8 µl of PCMV/PRV-FAM mix with 1.8 µl of pGAPDH-HEX mix as an internal control and 4.0 µl of extracted DNA. The reaction conditions for the PCMV/PRV real-time PCR were 2 min at 50°C for activation, then 10 min at 95°C, followed by 45 cycles of 15 s at 95 °C for denaturation and 60 s at 60 °C for annealing and elongation. As a positive control, a PCMV/PRV-specific gene block was used as described [55].

**Table 3:** Primers and probes used in this study. The PCMV real-time PCR was modified and performed as duplex PCR. PCR, polymerase chain reaction, PCMV, porcine cyteomegalovirus; pGAPDH, porcine glyceraldehyde-3-phosphate dehydrogenase; Fwd, forward primer; Rev, reverse primer; 6FAM, 6-carboxyfluorescein; BHQ, black hole quencher; HEX, hexachlorofluorescein

PCR assay	Accession number	Primer/probe	Sequence (5‘-3’)	Location (nucleotide number)	References
PCMV	AF268040.2	PCMV-Fwd	GTT CTG GGA TTC CGA GGT TG	5074-5093	[15, 54, 55]
		PCMV-Rev	ACT TCG TCG CAG CTC ATC TGA	5036-5116	
		PCMV-Probe	6FAM-CAG GGC GGC GGT CGA GCT C-BHQ	5095-5113	
pGAPDH	NM_001206359.1	pGAPDH-Fwd	ACA TGG CCT CCA AGG AGT AAG A	1083–1104	[53]
		pGAPDH-Rev	GAT CGA GTT GGG GCT GTG ACT	1188–1168	
		pGAPDH-Probe	HEX-CCA CCA ACC CCA GCA AGA GCA CGC-BHQ	1114–1137	

### Western blot analysis

Western blot analysis was performed as described previously [55, 56]. Briefly, for the detection of antibodies against PCMV/PRV, the Western blot assay described by Plotzki *et al*. [10] was used, but only the C-terminal fragment R2 of the gB protein of PCRV/PRV was used as an antigen. The R2 fragment of the gB of PCMV/PRV was produced in *Escherichia coli* BL21 cells using the pET16b expression vector encoding PCMV-R2 as described previously [10, 56]. Gene expression was induced by the addition of 1 mM isopropyl-β-D-thiogalactopyranoside (Roth, Karlsruhe, Germany), and when the cells were harvested, they were lysed in 10 mL of a solution containing 8 M urea, 0.5 M NaCl, 15 mM imidazole, and 20 mM Tris, pH 7.5. After centrifugation, the supernatant was applied to a HisTrap HP column installed on an Äkta Prime Plus system (both GE Healthcare, Chicago, Illinois, USA), washed, and eluted using a solution containing 6 M urea, 0.5 M NaCl, 500 mM imidazole, and 20 mM Tris, pH 7.5. The purified R2 protein was characterized by sodium dodecyl sulfate polyacrylamide gel electrophoresis (SDS-PAGE) as follows: The protein was dissolved in sample buffer (375 mM Tris-HCl, 60% glycerol, 12% SDS, 0.6 M dithiothreitol (DTT), 0.06% bromophenol blue) and denatured for 5 min at 95 °C prior to electrophoresis. SDS PAGE was carried out in a Mini-Protean Tetra Vertical Electrophoresis Cell (Bio-Rad Laboratories, Incs., Hercules, CA, USA) using a 12% polyacrylamide gel and a PageRuler prestained protein ladder (Thermo Fisher Scientific, Waltham, USA). The protein was transferred for 100 min to a polyvinylidene fluoride membrane (ROTI PVDF, 8989.1, Roth, Karlsruhe, Germany) by electroblotting at 100 mA, using the electroblotting device of peqlab Biotechnologie GmbH. The membrane was then blocked for 1 h at 4 °C in 5% non-fat dry milk (Roth, Karlsruhe, Germany) in PBS with 0.05% Tween 20 (Roth, Karlsruhe, Germany) phosphate-buffered saline (PBS-T) (blocking buffer). The membrane was cut into strips and incubated overnight at 4°C with sera diluted 1:300 in blocking buffer. The strips were then washed three times with 0.05% PBS-T for 10 minutes each. The strips were incubated for 1 h at room temperature in polyclonal goat anti-pig immunoglobulin G (IgG) Fc Secondary Antibody HRP (Invitrogen by Thermo Fisher Scientific, Waltham, USA) diluted 1:20,000 in blocking buffer followed by three washing steps for 10 minutes each. The signal appearing after incubation with ECL Western Blotting Substrate (Cytiva, Amersham) was detected using a FUSION-SL 3500 WL imaging device (peqlab Biotechnologie GmbH).

## Results

**Fig. 1:**
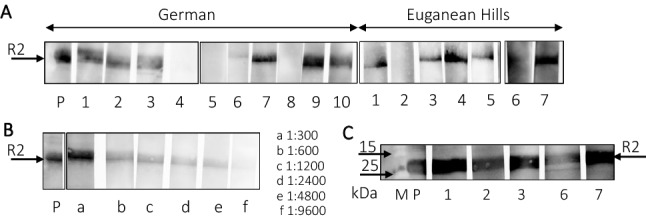
(A) Results of Western blot analysis of sera from German and Italian (Euganean Hills) wild boars using the recombinant fragment R2 of the gB of PCMV/PRV as an antigen. P, positive control. German pigs 4, 5, and 8 and Italian pig 2 were negative, and all other animals were positive. (B) Titration of the serum from a wild boar from the Euganean Hills against the recombinant fragment R2 of the gB of PCMV/PRV. P, positive control. (C) Repetition of the testing of the German sera 1, 2, 3, 6, and 7 using a higher concentration of polyacrylamide in the gel (17% instead of 12%) and a higher concentration of serum (a 1:150 dilution instead of 1:300).

All sera from Italian and German wild boars were screened for PCMV/PRV-specific antibodies using a Western blot assay that we established several years ago [10]. We used only the R2 fragment of the gB protein of PCMV because testing with this C-terminal recombinant fragment was more effective than using the N-terminal R1 fragment [10]. We were surprised to see such a high percentage of positive reactions (Tables 1 and 2) and such strong reactivity of the sera (Fig. 1). We used a 1:300 or 1:150 dilution of the sera but suggest that the number of positive sera may have been even higher with higher concentrations.

To analyze whether the DNA genome of PCMV/PRV could also be detected in the serum, DNA was isolated, and real-time PCR was performed using primers and a probe described by Mueller et al. [15], recognizing a conserved region in the DNA polymerase (DPOL) gene of PCMV. The real-time PCR was modified as a duplex real-time PCR using porcine GAPDH as a control [55, 56].

When we tested Italian wild boars using our Western blot assay, 54% of the animals were found to be positive. Using real-time PCR, 35% of the animals were positive, with the lowest C_t_ value indicating that the highest virus load was 30. There were differences in the positivity rates of animals from different geographical regions. The largest number of positive animals was found in the Veneto Alps (70% by Western blot, 80% by PCR) (Table 1), while the lowest percentage was found in the Friuli Venezia Giulia Alps (25% by both methods). In animals less than 22 months old, the detection rates by Western blot and real-time PCR were similar to the detection rates of the overall population (46% by Western blot and 30% by PCR). In animals more than 22 months old, no differences were observed in the detection rate by Western blot and PCR (in both cases 44%) (Table 1). A 1:4800 dilution, the serum from one wild boar from the Euganean Hills was still positive in a Western blot assay based on the R2 fragment of the gB of PCMV/PRV (Fig. 1B). This result is comparable with the situation in German slaughterhouse animals, where the highest positive dilution was 1:9600 [10].

When German wild boars were tested, a higher infection frequency was observed, with 82% of the animals positive by Western blot assay and 60% by real-time PCR (Table 2). In comparison with Italian wild boars, the C_t_ values were lower in many animals (as low as 22), indicating higher virus loads in these animals. In Wittstock, Döberitzer Heide, and Rauen-Zerwelin, 92, 100, and 100% of the animals were positive by Western blot assay, respectively. These results clearly show that the virus is broadly distributed. In addition, in all locations, the Western blot analysis detected more PCMV/PRV-positive animals than did real-time PCR.

## Discussion

Here, we describe a broad distribution of PCMV/PRV in European wild boars in Italy and Germany. Whereas 82% of German wild boars were found positive using a Western blot assay and 60% were positive using real-time PCR, 54% of Italian wild boars were found positive using Western blot and 36% were positive using real-time PCR. In Italy, the number of positive animals was higher in the Veneto Alps (70% by Western blot, 80% by PCR) compared to the Friuli Venezia Giulia Alps (25% by both methods). The Veneto Alps and the Friuli Venezia Giulia Alps have a similar density of animals and environmental and ecological features. An intermediate percentage of positive animals was found in the Euganean Hills, where animal density is much higher and the wild boar population is more isolated. These findings are in contrast with the prevalence of other viruses, such as PCV3 and HEV-3 [31, 57]. These viruses were detected more often in the Friuli Alps than in the in Euganean Hills Regional Park. In the Euganean Hills, a large number of individuals (approximately 2000 animals) are gathered in a small area (187 km^**2**^). This feature allows contact between animals and the transmission of viruses excreted by infected wild boars (31, 57). HEV-3 has been found in bile, liver, and feces, and wild boars can be considered excretors of this virus [57]. In contrast, PCMV/PRV, which exists in a latent state with a little excretion into the environment, is not distributed in this way. During latency PCMV/PRV can be found only in some organs, e.g., lung, liver, salivary gland, and kidney [12]. The frequency of reactivation is unknown, and additional studies are needed to get a deeper insight into this phenomenon.

There are indications that most infections occur in young animals, with possible transmission between mothers and piglets. The detection rate of PCMV/PRV by Western blot is slightly lower in pigs older than 22 months (44%) than the average detection rate for animals of all ages (54%). We recently showed that detection by PCR is easier in young piglets and more difficult in older animals when the virus is latent [56]. Why this was not the case in the present study (44% in animals older 22 months, 30% in younger animals) remains unclear. It is possible that some of the animals were otherwise ill, and this might have activated the virus.

Therefore, PCMV/PRV transmission is likely to occur during close sow-to-piglet contact after farrowing stress. The smaller number of virus-positive animals among older pigs also indicates that increased contact among individuals in a population over time probably does not lead to an increase in the infection rate. However, we cannot exclude the possibility that the antibody titer decreases with age or that infected animals die earlier than uninfected animals. PCMV/PRV is a herpesvirus that is able to establish a latent infection and is thus difficult to detect by PCR. We recently repeatedly tested a small group of landrace pigs for PCMV/PRV by real-time PCR and were able to detect the virus until week 17 [56]. Thereafter, the virus went into latency and the PCR tests from blood were negative. This result is in agreement with previous findings that the infectious titer, if any, is low in adult animals [12]. Testing of inbred miniature swine demonstrated that lung, liver, salivary gland, and kidney were PCR positive, but the gut tissue was consistently PCR-negative. However, the viral loads in the liver and salivary gland were below the threshold of quantification and were given an arbitrary value of less than 10 PCMV genome copies/mg of DNA. The kidney contained 38 genome copies/mg of DNA, and the lung contained 97 genome copies/mg of DNA [58].

As mentioned above, in young piglets, the virus can easily be detected by PCR. In adult animals, however, the detection of antibodies is the most effective approach to verifying virus infection, even if PCR yields negative results. For this reason, we used two different methods: (i) real-time PCR, which detects the viral DNA in an active infection, and (ii) a Western blot assay with viral antigen to screen for PCMV/PVR antibodies, indicating previous exposures and latent virus.

The first case of a pathologically and virologically diagnosed PCMV/PRV infection in a wild boar was reported in Japan [50]. In Northeastern Patagonia, Argentina (Buenos Aires and Río Negro Provinces), PCMV/PRV screening using a nested PCR assay on tonsil tissues from 62 free-living wild boars showed an overall infection rate of about 56% [51]. A significantly higher level (nearly 90%) was determined for animals less than 6 months old. In 2007, it was published that wild boars in some regions of Russia were carriers of Aujeszky's disease virus, porcine parvovirus, porcine circovirus type 2, lymphotropic herpesvirus 1, PCMV/PVR, *Mycoplasma hyopneumoniae*, and *Pasteurella multocida* [52].

The overall rate of PCMV/PVR infection in wild boars is in the same range as that reported for herds of domestic pigs in Asia, Europe, North America, and South America [8–10, 58]. In contrast, the seroprevalence of PCMV/PRV in pigs from Hunan province, China, was much higher, 96% (482/500), with the highest percentage found in breeding sows (97%) [59]. Either the infection rate in this province is indeed so high, or the antigen used in the ELISA contained bacterial contamination, and antibodies against these bacterial proteins may have caused false-positive results. A comparison of different study results is challenging, since different populations, tissues, and methods have been used for screening: either tonsils or tonsil swabs [8, 51] or sera [9, 10, this study]. When we analyzed 30 German slaughterhouse pigs, 52% were positive in a real-time PCR and 83% were positive in a Western blot assay using the same fragment (R2) of the gB protein of PCMV/PRV as used here [10]. In that study, the N-terminal fragment R1 of the gB protein of PCMV/PRV was also used as an antigen, but only 11% of the sera were positive, indicating that the R2 fragment is the better target of the immune system. For this reason, only the R2 fragment was used in the present study (Fig. 1).

There is evidence that PCMV/PVR infection occurs transplacentally when pregnant sows are inoculated experimentally with the virus [60, 61]. However, this was not observed in a study under natural conditions. Despite mother sows having PCMV/PRV DNA detectable in their spleen, neither transplacental infection in their offspring nor postnatal transmission was detected [12]. Furthermore, the authors showed that piglets that were delivered by caesarean section from PCMV/PRV-positive sows and subsequently barrier reared did not acquire PCMV/PVR [12].

Human cytomegalovirus (HCMV) affects up to three-quarters of all solid organ transplant human recipients [62]. As mentioned above, PCMV/PRV is not closely related to HCMV [1, 2]. However, human herpesviruses closely related to PCMV/PRV, HHV-6, and HHV-7 can cause common opportunistic infections in the post-transplantation period and have also been associated with transplant rejection in human solid organ transplant recipients [63–65].

This study on the prevalence of PCMV/PRV is important for xenotransplantation, since PCMV/PRV has been shown to drastically reduce the survival time of pig organs in non-human primates [17–21] and because PCMV/PRV contributed to the death of a patient in Baltimore [20]. However, there are sensitive detection methods and test strategies to prevent transmission of PCMV/PRV in future clinical trials [66, 67]. Our data indicate that facilities for raising pigs for xenotransplantation, which are PCMV/PRV-free, need to be protected from introduction of this virus not only by commercial pigs but also by wild boars. The pigs should be protected from contact with infected animals or materials from infected animals.

## Conclusion

PCMV/PRCV is broadly distributed in European wild boars in Italy and Germany. The number of PCMV/PRV-positive wild boars was found to be higher in Germany than in Italy. The Western blot assay detected more PCMV/PRV-positive animals than did the real-time PCR assay. Facilities for breeding pigs for xenotransplantation should be protected from contact with materials from infected commercial pigs as well as from wild boars.

## Supplementary Information

Below is the link to the electronic supplementary material.

Supplementary file1 (PPTX 187 KB)

Supplementary file2 (PPTX 102 KB)

## Data Availability

All data generated or analysed during this study are included in this published article.
