# Supplementary material for: Presence of porcine cytomegalovirus, a porcine roseolovirus, in wild boars in Italy and Germany
Source: Arch Virol. 2023 Jan 7;168(2):55. doi: 10.1007/s00705-022-05690-6 (PMC9825524; doi:10.1007/s00705-022-05690-6)
Supplement: Supplementary file 1 — Supplementary file1 (PPTX 187 KB) [file 705_2022_5690_MOESM1_ESM.pptx]

## Slide 1
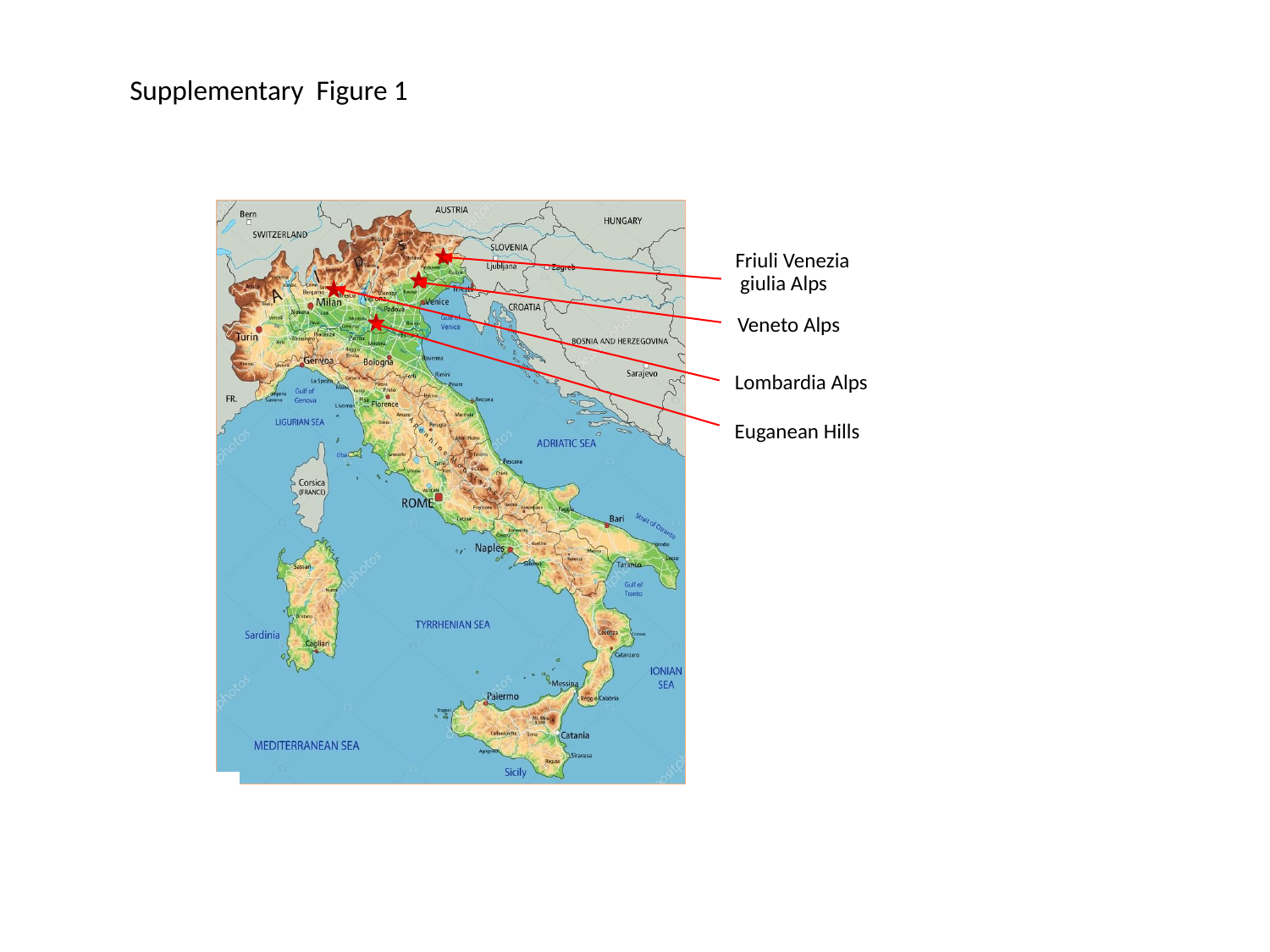

Supplementary Figure 1
© depositphotos
# Friuli Venezia giulia Alps
Veneto Alps
Lombardia Alps
Euganean Hills
