# Supplementary material for: Presence of porcine cytomegalovirus, a porcine roseolovirus, in wild boars in Italy and Germany
Source: Arch Virol. 2023 Jan 7;168(2):55. doi: 10.1007/s00705-022-05690-6 (PMC9825524; doi:10.1007/s00705-022-05690-6)
Supplement: Supplementary file 2 — Supplementary file2 (PPTX 102 KB) [file 705_2022_5690_MOESM2_ESM.pptx]

## Slide 1
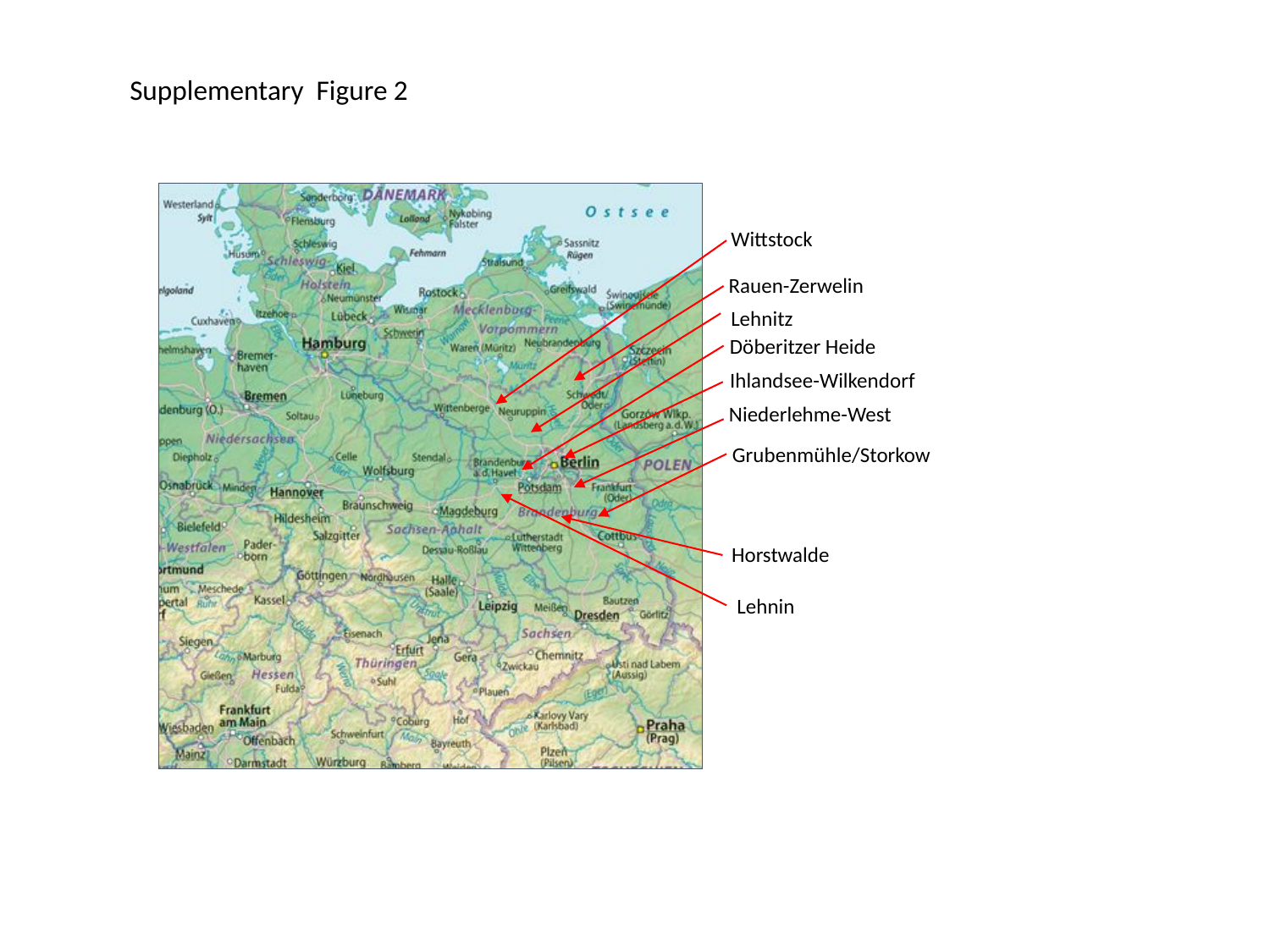

Supplementary Figure 2
# Wittstock
Rauen-Zerwelin
Lehnitz
Döberitzer Heide
Ihlandsee-Wilkendorf
Niederlehme-West
Grubenmühle/Storkow
Horstwalde
Lehnin
